# Structural and Sequence Similarities of Hydra Xeroderma Pigmentosum A Protein to Human Homolog Suggest Early Evolution and Conservation

**DOI:** 10.1155/2013/854745

**Published:** 2013-09-05

**Authors:** Apurva Barve, Saroj Ghaskadbi, Surendra Ghaskadbi

**Affiliations:** ^1^Division of Animal Sciences, Agharkar Research Institute, G. G. Agarkar Road, Pune 411 004, India; ^2^Department of Zoology, University of Pune, Ganeshkhind, Pune 411 007, India

## Abstract

Xeroderma pigmentosum group A (XPA) is a protein that binds to damaged DNA, verifies presence of a lesion, and recruits other proteins of the nucleotide excision repair (NER) pathway to the site. Though its homologs from yeast, *Drosophila*, humans, and so forth are well studied, XPA has not so far been reported from protozoa and lower animal phyla. Hydra is a fresh-water cnidarian with a remarkable capacity for regeneration and apparent lack of organismal ageing. Cnidarians are among the first metazoa with a defined body axis, tissue grade organisation, and nervous system. We report here for the first time presence of *XPA* gene in hydra. Putative protein sequence of hydra XPA contains nuclear localization signal and bears the zinc-finger motif. It contains two conserved Pfam domains and various characterized features of XPA proteins like regions for binding to excision repair cross-complementing protein-1 (ERCC1) and replication protein A 70 kDa subunit (RPA70) proteins. Hydra XPA shows a high degree of similarity with vertebrate homologs and clusters with deuterostomes in phylogenetic analysis. Homology modelling corroborates the very close similarity between hydra and human XPA. The protein thus most likely functions in hydra in the same manner as in other animals, indicating that it arose early in evolution and has been conserved across animal phyla.

## 1. Introduction 

 Nature has devised several strategies to cope with the constant assault of internal and external agents on the DNA of cells [1]. Among these, DNA lesions caused by ultraviolet (UV) rays or by chemicals that distort its helical structure are repaired by the nucleotide excision repair (NER) pathway [2]. NER involves cutting out a patch of DNA around the lesion and replacing it with undamaged nucleotides [3]. There are two sub-pathways of NER: global genome, that is, GG-NER and transcription coupled, that is, TC-NER. Components of the GG-NER subpathway scan the entire genome and repair helix-distorting lesions that they encounter. On the other hand, TC-NER is specialised for repairing DNA lesions that block transcription [1–3]. The principal players of NER are the xeroderma pigmentosum (XP) group of proteins [3]. The name XP is derived from the autosomal recessive disorder where affected individuals are sensitive to sun light, tend to have rough, pigmented skin, and show a high incidence of skin cancer. XP patients can be classified into seven complementation groups XP-A through XP-G, depending upon the specific gene that is affected. The protein encoded by each of these genes is responsible for carrying out a specific step of NER [3]. One of the first members to come into play during NER is XP group A (XPA) protein. This is a critical component of the pathway, without which repair cannot occur [4, 5].

XPA is a small, monomeric protein that acts in association with replication protein A (RPA) [6, 7]. It has no catalytic activity [7] but has a high affinity for damaged DNA [8]. According to the current model for the mechanism of damage recognition in NER [9], a complex of XPC and human homolog of Rad23 B (hHR23B) recognises and binds to possibly damaged DNA, while XPA-RPA confirms the damage and recruits other members to the site [9]. The important role of XPA is thus damage verification to ensure that repair occurs only at the point of lesion [9]. To facilitate its function, XPA bears a number of specific interaction domains [6, 10, 11], reviewed in [7]. In the course of NER, XPA interacts with DNA through its zinc-finger domain and binds with RPA, TFIIH, XPF-ERCC1, and so forth (reviewed in [7]). XPA protein is the rate limiting factor of NER [12], and its level in a cell is regulated by the ubiquitin ligase HERC2 [13]. 

 Though homologs of some NER genes are found in Archaea, *XPA* is present only among eukaryotes [14, 15]. UvrA, the damage recognition protein of the UvrABC system which is responsible for excision repair in bacteria, shows no sequence homology with XPA [16]. *XPA* gene from mammals like mouse and human is wellstudied [17]. In *Drosophila*, the *XPA* homolog is expressed in all developmental stages and is able to complement *XPA* deficient human cells, demonstrating evolutionary functional conservation of this gene [18]. Among the lower animal phyla, XPA was not identified in BLAST analysis of protozoan genomes, but partial sequence has been reported from the Cnidarian *Nematostella*. XPA and the NER pathway remain poorly studied in early-evolved animals.

 Cnidarians are amongst the first animals that possess a true tissue grade organization and nervous system and hence possess a unique position in the study of metazoan evolution [19]. The fresh-water hydrozoan hydra is a simple, diploblastic animal belonging to this phylum. It has a cylindrical body with a conical hypostome and tentacles on the apical side and a flat basal disc at the other end [20]. Under well-fed conditions, it continuously reproduces asexually by budding and has a very high regeneration capacity [20, 21]. Hydra has very peculiar tissue dynamics due to its three stem cell lineages that divide constantly to generate excess cells which move into buds or get sloughed off from the ends of body, keeping the size of the animal within a certain range [21]. Hydra is potentially immortal and does not senesce. Individual animals tracked for more than four years have survived without any signs of ageing [22]. Many genes and signalling pathways present in hydra have been shown to be conserved up to higher phyla, including vertebrates [19]. However, the NER pathway of this animal had thus far not been analyzed. We have recently identified and cloned parts of all seven XP genes from hydra for the first time. The complete CDS of hydra *XPF*, which encodes the 5′ endonuclease of NER, has been partially characterized [23]. Here, we report identification and cloning of the *XPA* homolog from *Hydra vulgaris *Ind-Pune [24]. We demonstrate that *XPA* is present in hydra, and its predicted protein possesses all the standard functional domains. This is the first report of characterization of XPA from one of the earliest metazoan phyla.

## 2. Materials and Methods

### 2.1. Isolation and Cloning of *XPA *from *H. vulgaris* Ind-Pune

 Amino acid sequence of human XPA protein (NP_000371.1) was used to carry out tBLASTx [25] analysis of the *H. magnipapillata *genome sequence at NCBI. Best scoring hydra sequences were selected and extracted from the output. The region suggested as the complete coding DNA sequence (CDS) of *XPA* was obtained and used for designing primers (Fwd: ATGGATGATAAAGTATCAGC; Rev: CTACATTTTTTCATATTTTAACTTA). Putative *XPA* sequence was amplified by PCR from the cDNA of *H. vulgaris* Ind-Pune, cloned into pGEM-T Easy vector (Promega), sequenced, and submitted to the NCBI GenBank database.

### 2.2. Sequence Alignment

*Hydra vulgaris* Ind-Pune *XPA* was analysed for homology using BLAST [25, 26] at the NCBI server to check the degree of similarity with *XPA* sequences from other species. The *H. vulgaris* Ind-Pune *XPA* CDS was translated *in silico *using ExPASy “Translate” software [27], and the amino acid sequence was analysed to determine various properties of the protein. 

### 2.3. Conserved Domain Analysis

The putative hydra XPA peptide sequence was further analysed using SMART [28, 29] to find the conserved domains within the protein. Multiple sequence alignment of XPA amino acid sequences from various animals was carried out using ClustalW program [30, 31]. XPA sequences from *Drosophila melanogaster *(BAA06690.1), *Xenopus laevis* (NP_001081354.1), *Gallus gallus *(NP_990184.1),* Mus musculus *(CAA52393.1), and *Homo sapiens *(NP_000371.1) were extracted from the NCBI database and used for alignment with *H. vulgaris* Ind-Pune XPA (AER00322.1). The alignment of human and hydra XPA sequences was screened for conserved amino acids, especially in regions known to form various functional domains of XPA. 

### 2.4. Homology Modelling

Swiss Model program at the ExPASy server [32, 33] was used for homology modelling of hydra XPA protein structure. Modelling was carried out by the automated mode and also using a specified structure entry from the protein data bank (PDB) as template. The generated models were compared with solved XPA structures from the database by superimposition using Deep View, the Swiss PDB Viewer software [34]. Superimposition was carried out using the “Iterative Magic Fit” tool of Deep View. Level of similarity between the model and solved structures was assessed by estimation of Root Mean Square Deviation (RMSD) value of the superimposed peptides. For both model and template, the number and position of residues used for RMSD calculation were determined automatically by the Iterative Magic Fit Tool. Modelling of XPA proteins from human (NP_000371.1), *X. laevis* (NP_001081354.1), and *D. melanogaster* (BAA06690.1) was carried out using automated mode at Swiss Model for comparison with the suggested structure of hydra XPA.

### 2.5. Phylogenetic Analysis

 Phylogenetic analysis of XPA proteins from various animals was carried out using MEGA 5.05 [35]. Following XPA sequences obtained from the NCBI protein database were used for this analysis: *H. vulgaris* Ind-Pune (AER00322.1), *Caenorhabditis elegans* (NP_492025.1), *D. melanogaster *(BAA06690.1), *Strongylocentrotus purpuratus *(XP_787964.2), *Ciona intestinalis *(XP_002128404.1), *Schistosoma japonicum *(CAX73181.1), *Danio rerio *(NP_956765.1), *X. laevis *(NP_001081354.1), *Anolis carolinensis* (XP_003227939.1), *G. gallus* (NP_990184.1),* M. musculus *(CAA52393.1), *H. sapiens *(NP_000371.1), and *Saccharomyces cerevisae *(NP_013928.1). The sequences were first aligned using the MUSCLE program in MEGA. Using these multiple sequence alignments, phylogenetic trees based on maximum parsimony (MP) [36], maximum likelihood (ML) [37], and minimum evolution (ME) [38] methods were constructed in MEGA. Bootstrap analysis [39] with 1000 replicates was performed for each tree.

## 3. Results and Discussion

### 3.1. Hydra XPA Shows High Sequence Similarity with XPAs from Vertebrates, Particularly Mammals

 BLAST analysis of the *H. magnipapillata* genome using human XPA protein as a query resulted in identification of a 774 bp predicted CDS showing similarity with *XPA* sequences from other animals. The complete *XPA* CDS was amplified from *H. vulgaris* Ind-Pune cDNA cloned and sequenced. BLAST analysis verified the identity of the sequence as *XPA*. This is the first report of cloning of *XPA* from hydra. In keeping with the (A + T) rich tendency of the hydra genome [40], hydra *XPA* CDS is composed of 65.5% (A + T). The nucleotide and putative protein sequences of *XPA* from *H. vulgaris* Ind-Pune have been deposited at the NCBI database under accession numbers JN411719.1 and AER00322.1, respectively.

 Putative hydra XPA protein sequence showed notable similarity with XPA from various animals in pBLAST analysis. *H. vulgaris* Ind-Pune XPA protein sequence is exactly identical to the predicted XPA sequence of *H. magnipapillata, *demonstrating the relatedness of the two species. Up to 52% identity and 70% similarity at the amino acid level was observed with XPA proteins from various other animals. The closest matches were found to be with homologs from many mammals like human, mouse, rat, dog, elephant, macaque, cattle, chimpanzee, and so forth, and other vertebrates like *Anolis*, *Xenopus*, *Danio,* and *Gallus*. This corroborates our earlier observations that hydra protein sequences often show greater similarity with their vertebrate homologs [23, 41]. The findings support the hypothesis of Kortschak et al. [42] that cnidarian genes are often more similar to vertebrate counterparts than to those in model invertebrates like *Drosophila*, indicating high complexity of cnidarian genomes and gene loss in model invertebrates. This is also in agreement with observations of Chapman et al. [40] who have observed a significant degree of synteny between hydra and other metazoan genomes. 

### 3.2. Hydra XPA Contains Conserved Nuclear Localization Signal (NLS)

**Figure 1:**
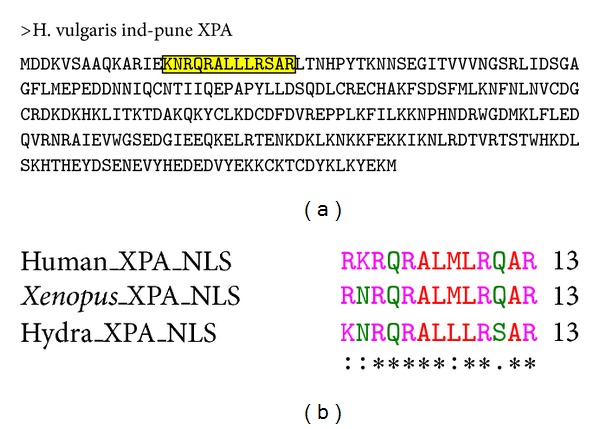
NLS in hydra XPA. (a) Amino acid sequence of hydra XPA with the 13 amino acids comprising NLS marked by box. (b) Alignment of NLS regions of XPA from human, *Xenopus,* and hydra shows that 9 residues out of 13 are completely conserved, while the rest are conservative substitutions.

 NLS is a tag which ensures that the protein is sorted into the nucleus. In the folded state, NLSs are present on the surface and are a feature of all proteins which function inside the nucleus like transcription factors, DNA replication, and repair proteins (reviewed in [43]). The NLS of human XPA lies in the 13 residue stretch from amino acid 30 to 42 [44] while that of *Xenopus *XPA extends from amino acid 29 to 41. XPA NLS is of the bipartite type [45], composed of two basic residues, a ten residue spacer, and another basic region consisting of three basic residues out of five. Comparison of hydra XPA sequence with *Xenopus* and human XPA showed that amino acids 15 to 27 of hydra XPA (Figure 1(a)) correspond to the NLS (Figure 1(b)). In the NLS, 9 out of 13 residues are fully conserved between these three species, while the remaining four are replaced by similar amino acids, indicating conservative substitutions (Figure 1(b)). Hydra XPA thus contains the necessary tag required for proper subcellular localization. 

### 3.3. Hydra XPA Shows Two Conserved Pfam Regions and Zinc-Finger, RPA-Binding, and ERCC1-Binding Domains

**Figure 2:**
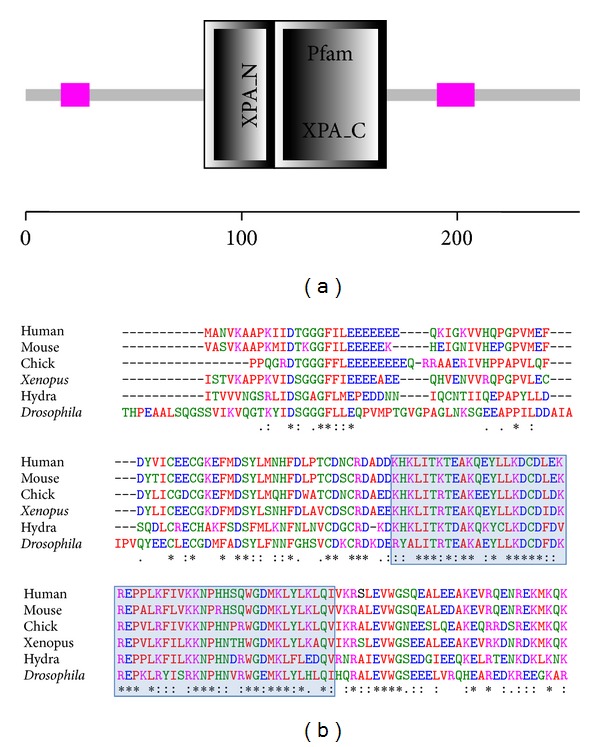
Conserved domains in hydra XPA. (a) Prediction of domains in hydra XPA by SMART: two Pfam domains XPA-N (amino acid 83 to 115) and XPA-C (amino acid 115 to 167) are present in the 257 amino acid long sequence. (b) Comparison of hydra XPA-N and -C domains with counterparts from other animals by multiple sequence alignment using ClustalW. Sequence of the XPA-C Pfam domain (box) is highly conserved across species. (Accession numbers of all sequences are given in Section 2).

SMART analysis of hydra XPA protein sequence revealed the presence of two conserved Pfam domains: XPA-N domain from amino acid 83 to 115 and XPA-C domain from 115 to 167 (Figure 2(a)). The e-value for prediction of both domains was extremely low (1.5*e* − 11 for XPA-N and 4.5*e* − 25 for XPA-C), indicating high degree of similarity between hydra XPA and respective domains from XPA proteins of other animals. Multiple sequence alignment of XPA proteins from various organisms by ClustalW showed that there is high level of conservation among the sequences, especially near the C-terminal. In the region comprising XPA-C, most amino acids are conserved indicating the critical nature of this Pfam domain (Figure 2(b), box).

**Figure 3:**
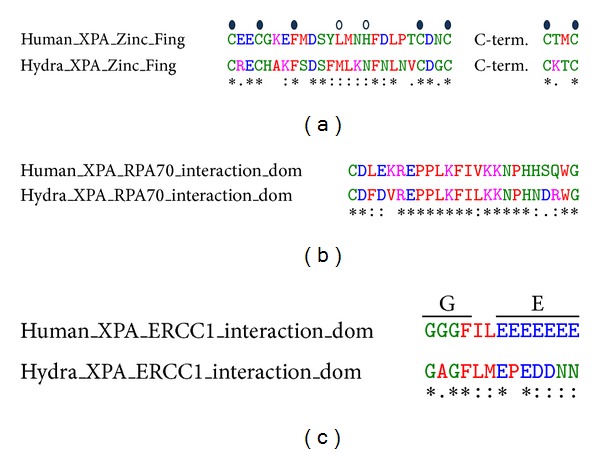
Comparison of various important regions of XPA from human and hydra. (a) Zinc-finger motif: seven amino acids (marked by filled circle) of the zinc finger including the CX2C motif near C-terminal are conserved between the two species, while two (marked by open circle) are conservative substitutions. (b) RPA70 binding region: seventeen out of 24 residues are conserved between human and hydra XPA while 5 more are similar. (c) Three out of 4 residues of G motif of ERCC1 binding region are conserved between human and hydra XPA, while the E motif is fairly variable.

 The XPA protein is noncatalytic but plays an important role in NER by binding to damaged DNA, recruiting several players to the site and positioning them for action (reviewed in [7]). Various regions of the XPA protein are involved in these interactions and hence are important for its functions. Zinc finger domain is one such region which is involved in DNA binding [46]. The zinc finger of human XPA has been localised to the XPA-N domain and consists of the sequence CX_2_CX_3_FX_4_LX_2_HX_5_CX_2_C and another CX_2_C motif near the C-terminal [46]. Comparison of this region with hydra XPA showed that the zinc finger motif is present in hydra XPA from amino acid number 87 to 111 (Figure 3(a), marked by circles). When respective regions of human and hydra XPA were compared, it was found that seven out of the nine important residues are totally conserved in hydra (Figure 3(a), filled circles), while two are replaced with conservative substitutions (Figure 3(a), open circles). All six critical cysteine residues including those in the CX_2_C motif at the C-terminal (Figure 3(a)) are present in hydra, indicating that hydra XPA contains a zinc finger motif.

 Another important functional domain of XPA is the replication-protein-A- (RPA-) binding domain. RPA is a heteromeric protein made up of three subunits, RPA70, RPA34, and RPA14 [47]. Interaction of XPA with RPA70 is critical for NER [6]. Using deletion mutants and pull-down assays, the domain for interaction with RPA70 has been shown to reside between residues 153 to 176 of human XPA [6]. Comparison of this 24 amino acid region from hydra and human XPA revealed that 17 residues out of 24 are completely conserved, while 5 are substituted with amino acids from the same group (Figure 3(b)). Thus, there is about 71% identity and more than 91% similarity in the RPA70-binding regions of human and hydra XPA proteins, indicating a high probability that XPA is able to bind to its putative RPA70 counterpart in hydra.

 XPA is known to interact with N-terminal region of the excision repair cross complementing protein-1 (ERCC1) [10, 11]. Li et al. [48] showed that this interaction is essential for NER. The site of interaction was localised to two conserved regions designated as G motif and E motif, located between amino acids 72 and 114 of human XPA. The G motif comprised of amino acids 72 to 75 of XPA in human is well conserved across species and critical for NER as demonstrated by the lack of complementation observed using ΔG-XPA [48]. In fact, the ΔG version of XPA is a dominant negative form that inhibits NER in wild type cells [48]. The E motif on the other hand, is present from residue 78 to 84 of human XPA and is conserved in vertebrates to varying extents but is not persistent outside the phylum [48]. 

Comparison of ERCC1 binding regions of hydra and human XPA showed that 3 out of four residues of the G motif are conserved in hydra, while the E motif is variable (Figure 3(c)). Analysis has shown that *ERCC1* gene arose in eukaryotes by duplication of *XPF,* and some of the domains acquired specialised properties of XPA- and DNA-binding by subfunctionalization [49]. Overall, observations suggest that the corresponding domains of ERCC1 and XPA may have coevolved to generate binding regions [15]. Hence, only the identification of XPA-binding domain in the ERCC1 protein from hydra will enable us to draw definite conclusions regarding the XPA-ERCC1 interaction in hydra.

It has been suggested that two cysteine residues in the C-terminal region of human XPA, C261, and C264 may be involved in interaction with TFIIH [50]. The two cysteines may form a disulphide bridge leading to a particular structure involved in interaction of XPA with TFIIH [7]. Both these cysteines are conserved in hydra XPA.

Hydra XPA thus appears to have all the domains necessary for interaction with its various partners during the NER process and hence seems likely to have a similar function in NER in hydra.

### 3.4. Predicted Structure of Hydra XPA Matches Solved Structures of Human XPA

**Figure 4:**
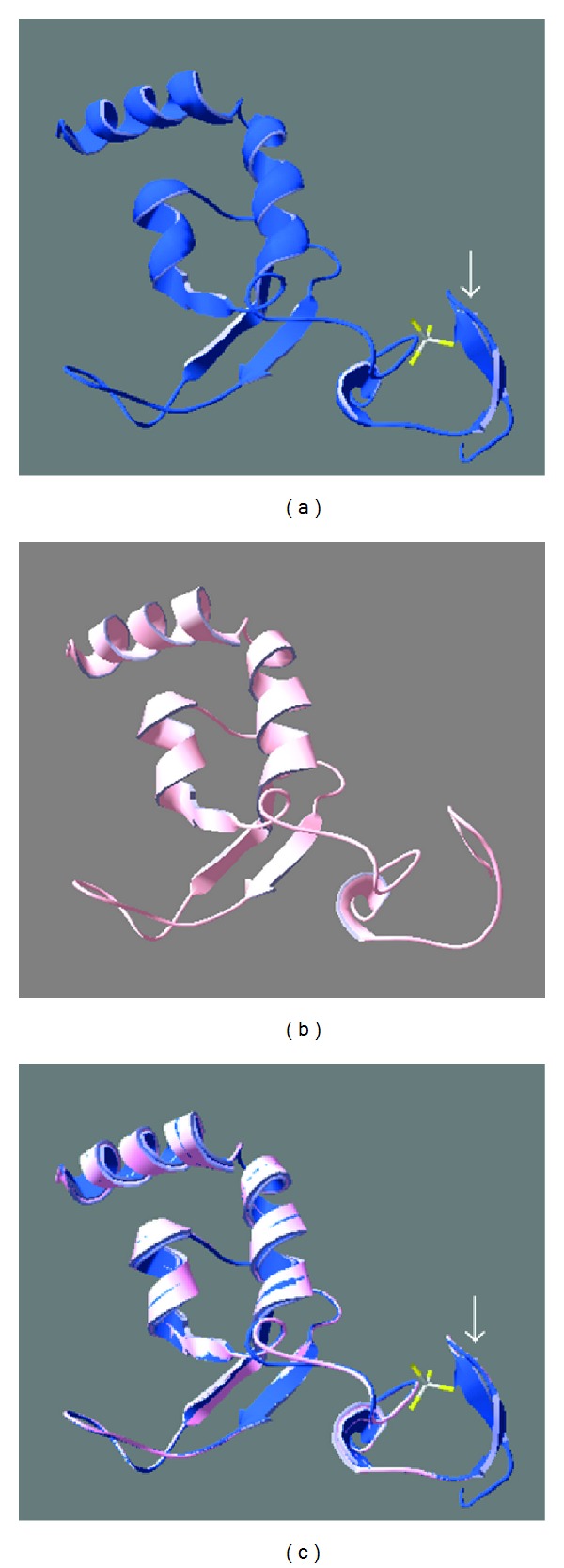
Predicted structure of hydra XPA based on NMR structure of human XPA. (a) Structure of DNA- and RPA-binding domains of human XPA (PDB id: 1XPA). (b) Predicted structure of hydra XPA modelled on 1XPA. (c) Superimposition of the two structures in SwissPDB Viewer. (An extra pair of beta sheets present in human XPA is marked by arrows in (a) and (c)).

**Figure 5:**
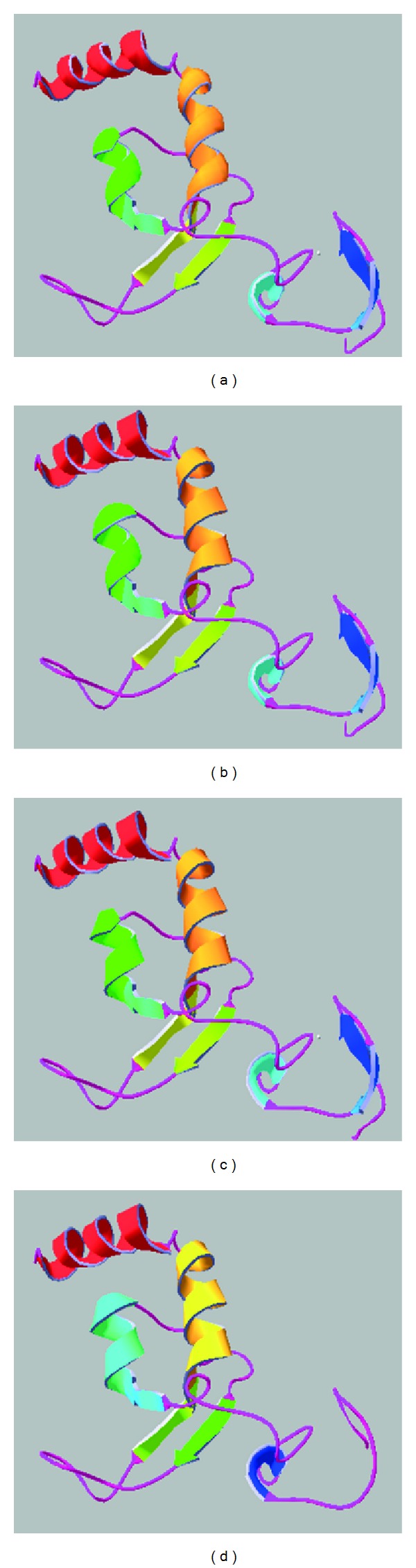
Putative structures of various XPA proteins. (a) Human, (b) *Xenopus*, (c) *Drosophila,* and (d) hydra XPA proteins generated by Swiss Model show very high similarity to each other, indicating similarity in function and conservation through evolution.

Structure for XPA domains involved in binding to DNA and RPA is solved [51, 52]. The secondary structures within the zinc-finger containing region and the C-terminal domain are well described with characterization of the DNA- and RPA-binding sites [51]. The NMR structures of this minimum binding domain of XPA are deposited in the protein data bank as 1XPA (Figure 4(a)) and 1D4U. 1XPA and 1D4U were used as templates for modelling hydra XPA in Swiss Model. The predicted structure of hydra XPA based on residues 86 to 191 shows very high similarity with the parent structures (Figure 4(b)). Superimposition of predicted hydra XPA structure with 1XPA showed a very good fit (Figure 4(c)). Except for a pair of beta sheets (arrows in Figures 4(a) and 4(c)) which are absent in hydra XPA, the two structures are almost identical. The second model of hydra XPA based on the PDB entry 1D4U covers the same residues and is also very similar to the template structure (not shown). The RMSD value of the superimposition of predicted hydra XPA structure on 1XPA was 0.13 Å, with all residues of the hydra XPA model used for calculation. This low RMSD value for the overlap of hydra and human XPA structures emphasizes the resemblance between the two proteins. Moreover, when XPA protein sequences from human, *Xenopus,* and *Drosophila* are modelled in automated modelling program of Swiss Model, they also use 1XPA as default template and yield very similar models (Figure 5), suggesting that based on similarity in structure, hydra XPA probably functions in a manner similar to its human and other homologs.

XPA is thus conserved across species at not just sequence but also at structure level, indicating that this protein arose early in evolution and has a critical function.

### 3.5. Hydra XPA Clusters with Higher Animal Homologs in Phylogenetic Analysis

**Figure 6:**
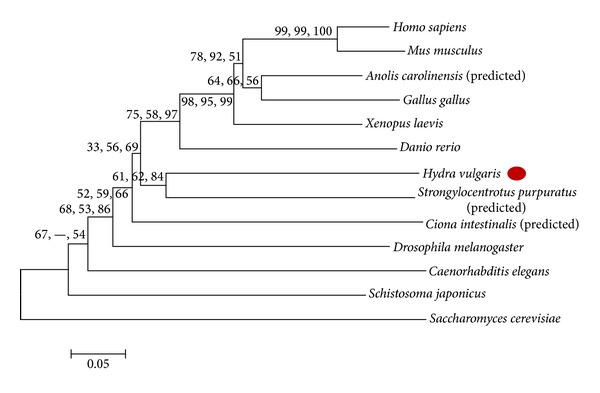
Phylogenetic analysis of XPA proteins. A representative ME tree with branch lengths in units of number of amino acid substitutions per site is shown. Bootstrap values for 1000 replicates for MP/ML/ME trees shown next to branch points. Positions containing gaps and missing data were eliminated. MP tree was obtained using the close-neighbour-interchange method with initial trees obtained by random addition of sequences. Initial tree for ML was constructed using BioNJ method with nearest neighbour interchange used for tree topology search. Discrete gamma distribution with 4 categories was used to model evolutionary rate differences among sites. For ME tree, evolutionary distances were computed using p-distance method and tree was searched using close-neighbour-interchange algorithm.

Phylogenetic trees using MP, ML, and ME (Figure 6) methods were constructed for XPA protein. The bootstrap consensus trees inferred from 1000 replicates were considered for analysis for each method. The percentage of trees in which the associated taxa clustered together in the bootstrap test with 1000 replicates is shown next to the branch points. Rad14, the *S. cerevisae* homolog of XPA, was used as outgroup for building the trees. Trees derived by all three methods showed a similar pattern. All the vertebrate XPAs consistently grouped together indicating their close relatedness, while echinoderm (*Strongylocentrotus*) XPA was found to be closely related to hydra XPA. This cluster of hydra and echinoderm XPA consistently grouped with the chordate cluster (Figure 6). XPA protein from the urochordate* Ciona *is the next closest relative of hydra-echinoderm-vertebrate group while invertebrates including* Drosophila* and* C. elegans* lie outside this branch.

The *XPA* gene has been identified in many organisms across the animal kingdom but has not yet been reported from the Archaea [14]. In fact, *XPA* seems to be absent in the single-celled eukaryote, *Plasmodium* [14], and a search for *XPA* among other protozoa during the present study also did not yield any hits. This indicates that *XPA* arose in more complex eukaryotes [14]. XPA is not yet reported from many members of lower taxa. Present study is the first report of identification and characterization of complete coding sequence of *XPA* from a cnidarian though a partial *XPA* mRNA from *Nematostella* is present in the database. Hence this study has special significance in analysis of evolution of XPA protein with respect to the unique evolutionary position of cnidaria. The similar pattern observed in trees generated by various methods and good bootstrap values at most nodes indicate the reliability of the current representation of phylogenetic relationship among XPA proteins. The present analysis showed that the yeast homolog of XPA, Rad14, is distant from its counterparts in the animal kingdom. Hydra XPA is closely related to echinoderm XPA and groups with the deuterostomes rather than protostomes. This matches with our observations regarding another NER gene, *XPF*, where the cluster of early metazoan XPFs consistently grouped with the chordate-echinoderm cluster [23]. Since XPA sequences from other early metazoan members are not available at present, conclusions drawn on the basis of hydra XPA show that the observations of Kortschak et al. [42] and Chapman et al. [40] regarding the similarity between cnidarian and bilaterian lineages despite early divergence hold true for XPA as well. This is also a further confirmation of the observations from BLAST analysis that hydra XPA protein is closely related to its vertebrate homologs.

It is quite well known that in addition to classic XP symptoms, some XP-A patients also suffer from several neurological problems [53, 54]. Although mechanism of action and function of XPA in NER has been worked out, the neurological abnormalities in XP-A patients remain poorly explained [55]. It is possible that apart from NER, XPA also plays a role in development or functioning of the nervous system. Hydra belongs to phylum Cnidaria, the first metazoan phylum to evolve nerve cells. It has a very simple nervous system consisting of a nerve net of interconnected neurons [19, 21]. It is thus an ideal model to investigate the role of XPA in nervous system function. The present analysis has shown that hydra XPA is very similar to its human and other vertebrate homologs, further adding to its value as a model to study the role of XPA in nervous system. Also, the *XPA* gene has not so far been identified protozoans, but first appears around the transition from unicellular to multicellular organisms. This observation makes the analysis of a second function of XPA even more interesting from the evolutionary perspective. This is the first report of identification and characterization of this gene from hydra. The stage is now set for further, in-depth enquiry of various functions of XPA in this animal, which, in turn is likely to provide vital clues on its evolutionary significance.

## 4. Conclusions

The presence of *XPA*, a gene from the NER pathway, is demonstrated in hydra for the first time. As a representative of the phylum that shows true tissue-grade organization and a nervous system for the first time in evolution, hydra holds a unique position in the study of evolution of complex pathways. Our results show that hydra XPA sequence and domains are very similar to other characterized XPA proteins. Homology modelling has shown that hydra XPA is likely to attain a structure very similar to human XPA in the folded state, further hinting that hydra XPA functions like other XPA proteins. Our data point to conservation of XPA at sequence, domain, structure, and possibly functional level across animal phyla. Given that *XPA*-like genes have not been reported so far from either Archaea or even unicellular eukaryotes, this report of presence of *XPA *in an early eumetazoan like hydra is noteworthy. Since XPA is a component of the NER, our results indicate that NER is an evolutionarily ancient and crucial pathway which is important for the sustained life of organisms. Further analysis of various aspects of *XPA* in hydra will be of great value to understand the evolution of not only NER in animals, but possibly also the evolution of the nervous system.
